# Stability of temperate coral *Astrangia poculata* microbiome is reflected across different sequencing methodologies

**DOI:** 10.3934/microbiol.2019.1.62

**Published:** 2019-03-01

**Authors:** Dawn B. Goldsmith, Zoe A. Pratte, Christina A. Kellogg, Sara E. Snader, Koty H. Sharp

**Affiliations:** 1 St. Petersburg Coastal and Marine Science Center, U.S. Geological Survey, St. Petersburg, FL 33701, USA; 2 School of Biology, Georgia Institute of Technology, Atlanta, GA 30332, USA; 3 Cherokee Nation Technologies, contracted to U.S. Geological Survey, St. Petersburg, FL 33701, USA; 4 Department of Biology, Marine Biology and Environmental Science, Roger Williams University, Bristol, RI 02809, USA

**Keywords:** *Astrangia poculata*, coral, microbiome, bacteria, archaea, microbes, clone library, Sanger sequencing, 16S rRNA

## Abstract

The microbiome of the temperate coral *Astrangia poculata* was first described in 2017 using next-generation Illumina sequencing to examine the coral's bacterial and archaeal associates across seasons and among hosts of differing symbiotic status. To assess the impact of methodology on the detectable diversity of the coral's microbiome, we obtained near full-length Sanger sequences from clone libraries constructed from a subset of the same *A. poculata* samples. Eight samples were analyzed: two sets of paired symbiotic (brown) and aposymbiotic (white) colonies collected in the fall (September) and two sets collected in the spring (April). Analysis of the Sanger sequences revealed that the microbiome of *A. poculata* exhibited a high level of richness; 806 OTUs were identified among 1390 bacterial sequences. While the Illumina study revealed that *A. poculata*'s microbial communities did not significantly vary according to symbiotic state, but did vary by season, Sanger sequencing did not expose seasonal or symbiotic differences in the microbiomes. Proteobacteria dominated the microbiome, forming the majority (55% to 80%) of classifiable bacteria in every sample, and the five bacterial classes with the highest mean relative portion (5% to 35%) were the same as those determined by prior Illumina sequencing. Sanger sequencing also captured the same core taxa previously identified by next-generation sequencing. Alignment of all sequences and construction of a phylogenetic tree revealed that both sequencing methods provided similar portrayals of the phylogenetic diversity within *A. poculata*'s bacterial associates. Consistent with previous findings, the results demonstrated that the *Astrangia* microbiome is stable notwithstanding the choice of sequencing method and the far fewer sequences generated by clone libraries (46 to 326 sequences per sample) compared to next-generation sequencing (3634 to 48481 sequences per sample). Moreover, the near-full length 16S rRNA sequences produced by this study are presented as a resource for the community studying this model system since they provide necessary information for designing primers and probes to further our understanding of this coral's microbiome.

## 1. Introduction

*Astrangia poculata*, the northern star coral, is a temperate scleractinian coral with a wide geographic range. It has been documented on the Atlantic Coast of the United States from Maine to Florida, as well as the Gulf Coasts of Florida, Louisiana and Texas [1],[2]. This ahermatypic coral also grows in the eastern Atlantic off the west coast of Africa [1],[2], and has been documented at depths ranging from 0 to 263 m [1].

One of the distinguishing features of *A. poculata* is its facultative symbiosis with members of the endosymbiotic dinoflagellates *Breviolum psygmophilum* (formerly *Symbiodinium psygmophilum*) [1],[3]–[6]. Polyps with low dinoflagellate density (aposymbiotic) appear white and translucent [7], while symbiotic polyps of *A. poculata* are brown, reflecting the pigments of the algae that reside in the coral [5],[7]. Both symbiotic states can coexist within a single colony, a state referred to as “mixed”, resulting in a mottled appearance [1],[5].

Because symbiotic, aposymbiotic, and mixed colonies of *A. poculata* occur naturally, the species is an ideal organism to study microbial community interactions associated with symbiotic state. The first study to describe the bacterial and archaeal associates of *A. poculata* used Illumina sequencing and found a significant influence of season on alpha diversity within each sample's microbial community [5]. However, there was no significant difference between microbiomes of brown versus white colonies; the microbiome of *A. poculata* remained stable regardless of the coral's symbiotic state [5]. Six phylogenetic classes dominated the microbiome, including three classes from the Proteobacteria phylum (Gamma-, Delta-, and Alphaproteobacteria), Flavobacteriia, Cytophagia, and the archaeal class Thaumarcheota.

Moreover, Sharp et al. discovered that four bacterial operational taxonomic units (OTUs) and one OTU from the Thaumarchaeota class (genus *Nitrosopumilus*) appeared in 100% of their 72 *A. poculata* samples [5]. Identifying core microbiome members is crucial for understanding symbiont-host ecology [8]. Core bacteria may perform critical functions for the coral, potentially related to the health or nutrition of the host [8],[9]. The dominant members of a microbiome are not always the same as the core members; rather, core members, though ubiquitous, may comprise only a small portion of the host's microbial community [10]. Identifying core members of *A. poculata*'s microbiome is a key step in researching the interactions between the host and its microbial community [8].

Currently, this coral species is considered a model system for examining coral-microbial interactions (https://kotysharp.weebly.com/astrangia-workshop.html). However, the ability to develop primers and probes to more specifically target key microbial groups has been hindered by the lack of full length 16S rRNA sequences, since sequences produced by the Illumina platform are of insufficient length (approximately 250 base pairs) for the design of primers and probes.

The first goal of this study was to determine whether sequencing methodology affected the observed diversity of the *A. poculata* microbiome, including detection of the core microbiome. The second goal was to create a resource for the research community by generating a dataset of longer sequences that are better suited for the development of probes and primers.

## 2. Materials and methods

### 2.1. Sample collection and storage

Paired brown (symbiotic) and white (aposymbiotic) samples of *A. poculata* were collected as described in Sharp et al. [5]. Briefly, samples were collected from Narragansett Bay (Fort Wetherill State Park, Jamestown, RI) in September 2015 and April 2016 by SCUBA from depths of 1–5 m (Table 1). Paired colonies were selected such that the brown and white members of the pair were no more than 10 cm apart. Samples were immediately brought to the surface, frozen in liquid nitrogen, and held at −80 °C until DNA extraction.

**Table 1.** *A. poculata* sampling dates, symbiont status, and number of clone library bacterial sequences obtained for each sample. All samples were collected via SCUBA at Fort Wetherill State Park, Jamestown, RI (41° 28′ 40″ N, 71° 21′ 34″ W) from a depth of 1–5 m.

Sample	Date of collection	Symbiont status	Number of bacterial sequences obtained before filtering	Number of bacterial sequences remaining after filtering
FW1B4_CL	Sept. 9, 2015	Brown (symbiotic)	480	81
FW1W4_CL	Sept. 9, 2015	White (aposymbiotic)	480	217
FW1B8_CL	Sept. 9, 2015	Brown (symbiotic)	480	246
FW1W8_CL	Sept. 9, 2015	White (aposymbiotic)	480	221
FW3B7_CL	April 29, 2016	Brown (symbiotic)	480	46
FW3W7_CL	April 29, 2016	White (aposymbiotic)	480	326
FW3B9_CL	April 29, 2016	Brown (symbiotic)	480	159
FW3W9_CL	April 29, 2016	White (aposymbiotic)	480	94

### 2.2. DNA extraction

DNA was extracted from each sample of *A. poculata* as described in Sharp et al. [5]. Briefly, the PowerSoil DNA Isolation Kit (QIAGEN, Germantown, MD) was used according to the manufacturer's protocol to extract DNA from a fragment of each sample comprising mucus, tissue, and skeleton.

### 2.3. DNA amplification and quantification

*Bacterial primers*: DNA from each sample was amplified by PCR using primers 8F (5′-AGA GTT TGA TCC TGG CTC AG) and 1492R (5′-GGT TAC CTT GTT ACG ACT T) to target the 16S rRNA gene [11],[12]. Each 25-µL reaction contained 12.5 µL AmpliTaq Gold 360 Master Mix (Applied Biosystems, Foster City, CA), 0.4 µM concentration of each primer, and 10 µL of template DNA. The reaction conditions consisted of 15 min of initial denaturation at 95 °C, 30 cycles of (i) 1 min denaturation at 95 °C, (ii) 1 min annealing at 54 °C, and (iii) 2 min extension at 72 °C, and 10 min of final extension at 72 °C. Amplicons were visualized on a 1.5% agarose gel, then extracted from the gel using the QIAquick Gel Extraction Kit (QIAGEN, Germantown, MD) according to the manufacturer's instructions. Gel-extracted amplicons were quantitated using a Qubit dsDNA HS Assay Kit (Thermo Fisher Scientific, Waltham, MA) on a Qubit 3.0 fluorometer according to the manufacturer's instructions.

*Archaeal primers*: DNA from two of the samples (FW1B8 and FW1W8) was also amplified using primers 21F (5′-TTC CGG TTG ATC CYG CCG GA) and 958R (5′-YCC GGC GTT GAM TCC AAT T) in order to amplify the 16S rRNA gene from Archaea [13]. Each 25-µL reaction contained 12.5 µL AmpliTaq Gold 360 Master Mix (Applied Biosystems, Foster City, CA), 0.4 µM concentration of each primer, and 10 µL of template DNA. The reaction conditions consisted of 15 min of initial denaturation at 95 °C, 30 cycles of (i) 95 °C for 1.5 min, (ii) 55 °C for 1.5 min, and (iii) 72 °C for 1.5 min, and 10 min of final extension at 72 °C [13]. Amplicons were visualized, extracted, and quantitated as described above for the bacterial amplicons.

### 2.4. Cloning and sequencing

Amplicons were cloned into the pDrive vector using the PCR Cloning Plus kit (QIAGEN, Germantown, MD) and used to transform competent cells. After M13 screening, inserts in positive transformants were sequenced by the Clemson University Genomics Computational Laboratory (Clemson, SC) using Sanger sequencing on a 3730xl DNA Analyzer (Applied Biosystems, Foster City, CA).

### 2.5. Sequence processing and deposition

Vector and ends were trimmed from the sequences using Geneious (version 11.1.4; Biomatters Ltd., Auckland, NZ). Using QIIME version 1.9.1 [14], all sequences less than 50 bp were removed. Greengenes (version 13_8) [15]–[17] was used through QIIME to perform a chimera check with usearch61 [18], and to classify taxonomy using an open reference algorithm with a 97% similarity threshold [18]. Singletons were retained, while all other default parameters were used. After chimeric, unclassified, chloroplast, and mitochondrial sequences were removed, 996 bacterial OTUs and 18 archaeal OTUs remained. Upon submission of the sequences to NCBI's GenBank, GenBank's implementation of version 10 of usearch (64-bit version) using the uchime2_ref command in high confidence mode [19] uncovered 190 additional chimeras among the bacterial sequences. These sequences were removed, resulting in a data set of 806 bacterial OTUs (Table S1) and 18 archaeal OTUs (Table S2). Sequences representing each bacterial OTU have been deposited in GenBank under accession numbers MK175495 to MK176300. Sequences representing each archaeal OTU have been deposited in GenBank under accession numbers MH915525 to MH915542. The sequences are also available as part of a USGS data release [20].

### 2.6. Sequence analysis

The previously published unrarefied OTU table produced by Illumina next generation sequencing technology [5] was modified for the purposes of this study. All samples not analyzed in this study were removed from the Illumina table, as well as archaeal OTUs, as the current study used separate primers for archaea and bacteria, and therefore could not include archaea in quantitative analyses. After these modifications, 7687 OTUs remained. Finally, the modified next-generation OTU table was rarefied to the smallest number of sequences remaining among the eight samples (3516 sequences per sample among 4400 OTUs) using QIIME2 (version 2018.6) [21]. The OTU table generated from the clone library sequences was also rarefied in QIIME2 to the smallest number of clone library sequences remaining in a sample after filtering (46 sequences among 282 OTUs). These rarefied OTU tables were used as the basis for calculating the Shannon diversity index in R using the vegan package [22]. The Shannon index was also calculated for the full (unrarefied) clone library data for comparison purposes. Greengenes (version 13_8, through QIIME) was used for taxonomic classification of the clone library sequences for consistency with those assigned for the next-generation sequences in Sharp et al. [5]. Community composition was assessed using the unrarefied OTU tables at the class level. Each class comprising at least 2.5% of any sample was identified individually, while all others were grouped as Other. Relative abundance column graphs were prepared in R using base graphics [23].

### 2.7. Beta diversity

Beta diversity was analyzed using PRIMER 7 (version 7.0.13; PRIMER-E [24]) with PERMANOVA+. The bacterial OTU abundance tables for each data set (after rarefaction of the next-generation bacterial data) were square-root transformed and then used to calculate Bray-Curtis similarity [25]. Permutational multivariate analysis of variance (PERMANOVA) [26] was used to determine whether there was a significant difference between bacterial communities originating from samples of different seasons (fall versus spring) or symbiotic state (brown colonies versus white colonies).

### 2.8. Phylogenetic analysis

Aligned sequences consisted of one representative sequence for each OTU that contained more than two sequences in the unrarefied table of next-generation sequences (3630 OTUs), and one representative sequence for each OTU in the full OTU table of clone library sequences (806 OTUs). Sequences were aligned with SSU-ALIGN (release 0.1.1) using default parameters [27]. SSU-ALIGN also generates confidence estimates that each nucleotide was correctly aligned, based on the parameters of the model used for alignment. Based on those estimates, the program was used to mask the parts of the alignment that were most likely to contain errors, and the masked alignment was used for construction of a phylogenetic tree by FastTree (version 2.1.10) with default parameters [28]. The tree was visualized using the ape package [29] in R.

### 2.9. Analysis of shared OTUs

Core next-generation bacterial OTUs that were captured by clone library sequences were aligned with BLASTN [30] using the next-generation sequence as the query, all clone library OTU sequences as the subject, the option to align two or more sequences, and all default parameters except for setting the maximum number of target sequences to 1000. BLASTN was also used to align archaeal OTUs that appeared both in clone libraries and in next-generation sequences, using the next-generation sequence as the query, the clone library OTU sequence as the subject, the option to align two or more sequences, and all default parameters.

## 3. Results

Bacterial 16S rRNA sequences obtained from the eight coral colonies totaled 3880. After filtering the data to remove sequences less than 50 bp long, chimeras, chloroplast and mitochondria sequences, and sequences that could not be classified, 1390 bacterial sequences remained (Table 1), classified into 806 OTUs (Table S1). One-hundred ninety-two archaeal 16S rRNA sequences were obtained from two of the colonies (FW1B8 and FW1W8) separately, using archaea-specific primers. After filtering, 189 archaeal sequences remained, which were classified into 18 OTUs (Table S2).

### 3.1. Shannon diversity index

The Shannon diversity index considers both richness and evenness of the communities [31]. When calculated from the rarefied bacterial clone library OTU table, the Shannon index for the clone library sequences ranged from 3.39 to 3.80 (Table 2). Analysis of the full set of bacterial clone library data (unrarefied) produced Shannon indices ranging from 3.58 to 5.21. In contrast, the next-generation bacterial sequences generated Shannon indices ranging from 4.70 to 6.53 (Table 2). Hutcheson's t-test indicated that the diversity of the next-generation sequences was significantly greater than the diversity of the clone library sequences (Table S3) [32].

**Table 2.** Shannon diversity indices of bacterial communities associated with each *A. poculata* sample. Indices for clone library bacterial communities are presented before and after rarefaction of the abundance table for comparison. Clone libraries were rarefied to 46 bacterial sequences per sample. The next-generation sequences were rarefied to 3516 sequences per sample. Source of next-generation sequence data is Sharp et al. [5].

Sample	Season	Symbiont status	Shannon index, next-generation bacterial community	Shannon index, clone library bacterial community,before rarefaction	Shannon index, clone library bacterial community, after rarefaction
FW1B4	Fall	Brown (symbiotic)	5.42	3.85	3.50
FW1W4	Fall	White (aposymbiotic)	6.12	4.96	3.74
FW1B8	Fall	Brown (symbiotic)	6.53	5.20	3.71
FW1W8	Fall	White (aposymbiotic)	6.41	5.21	3.80
FW3B7	Spring	Brown (symbiotic)	5.29	3.58	3.58
FW3W7	Spring	White (aposymbiotic)	5.66	4.15	3.41
FW3B9	Spring	Brown (symbiotic)	4.70	3.98	3.39
FW3W9	Spring	White (aposymbiotic)	6.29	4.36	3.67

### 3.2. Community composition

**Figure 1.**
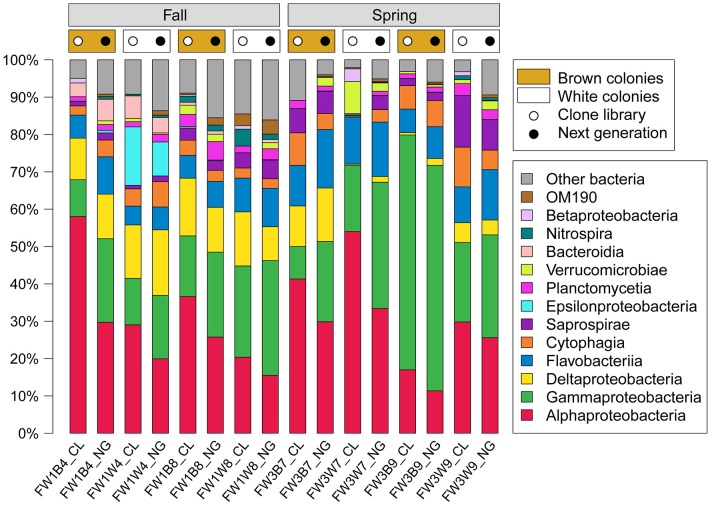
Composition of *A. poculata* bacterial communities as determined by clone library and next-generation sequences. Each class comprising at least 2.5% of any sample was identified individually, while all others were grouped as Other Bacteria. Source of next-generation sequence data is Sharp et al. [5].

The five classes that formed the largest average components of the samples' bacterial communities as represented by the clone libraries (Alphaproteobacteria, Gammaproteobacteria, Deltaproteobacteria, Flavobacteriia, and Cytophagia) are the same top five classes identified through the next-generation sequence analysis [5]. Proteobacteria formed the majority of bacteria in every sample (Figure 1). In particular, Alphaproteobacteria comprised the largest component of six of the eight samples analyzed by clone libraries, and ranged from 17% to 58% of the bacterial community in all clone library samples. These amounts exceeded the portion of the bacterial communities made up of Alphaproteobacteria in the next-generation sequences (11% to 30%; Figure 1). Gammaproteobacteria, in contrast, made up a smaller portion of the clone library sequences, but a larger portion of the next-generation sequences in all but one of the samples (FW3B9, which consisted of 62.9% Gammaproteobacteria in the clone library sequences, but 60.4% Gammaproteobacteria in the next-generation sequences). In the clone libraries, Gammaproteobacteria constituted 9% to 63% of the bacterial communities, while in the next-generation sequences Gammaproteobacteria comprised 17% to 60% of the bacterial communities.

### 3.3. Beta diversity

The Bray-Curtis similarity matrices for the clone library data (Table S4) and the next generation data (Table S5) indicate that the bacterial communities as determined by next generation sequencing display higher between-sample similarity than the between-sample similarity exhibited by the clone library bacterial communities. The clone library sequences displayed no significant difference among bacterial communities originating from brown versus white colonies (PERMANOVA, F = 0.99707, p = 0.55). The same result was observed among the next generation sequences, using an OTU table based on the bacterial subset of those sequences [5] (PERMANOVA, F = 0.99199, p = 0.59). PERMANOVA analysis similarly demonstrated no significant difference between fall and spring bacterial communities in the clone libraries (F = 1.0941, p = 0.49). In contrast, using an OTU table based on the bacterial subset of next generation sequences [5], PERMANOVA analysis revealed that bacterial communities differed by season (F = 2.6123, p = 0.03).

### 3.4. Phylogenetic analysis

**Figure 2.**
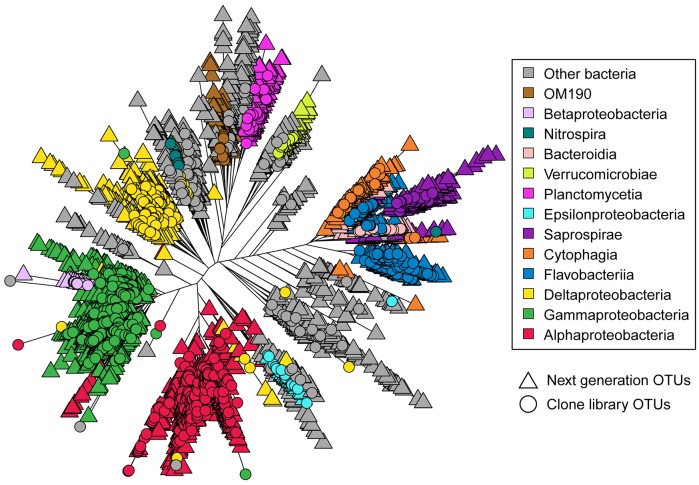
Phylogenetic tree representing bacterial OTUs from clone libraries and next-generation sequencing. OTUs from next-generation sequencing are displayed if the OTU contained more than two sequences in the unrarefied OTU table (3626 OTUs). Source of next-generation sequence data is Sharp et al. [5].

Although the next generation bacterial sequences yielded many more OTUs than the clone library sequences (7687 OTUs versus 806 OTUs), phylogenetic analysis revealed substantial overlap among the two sets of OTUs (Figure 2). In order to visualize the phylogenetic placement of the clone library bacterial OTUs without the more abundant next generation bacterial OTUs obscuring them, the leaves representing next generation OTUs were plotted first. The leaves representing clone library OTUs were plotted next, in order to overlay the next generation OTUs. In some classes (e.g., Cytophagia, OM190, Verrucomicrobiae, Deltaproteobacteria), branches of next-generation OTUs extend further than the branches of clone library OTUs, indicating that next-generation sequencing uncovered OTUs in those classes that are slightly more divergent than the clone library OTUs. Only a small number of branches, representing only a few OTUs, display next-generation sequences without clone library sequences overlaid.

### 3.5. Analysis of shared OTUs

Sharp et al. identified four bacterial OTUs that appeared in the microbiome of every coral sample [5]. All four of those next-generation OTUs were captured by clone library sequencing (Table 3). The OTUs were classified by Greengenes as members of the *Inquilinus* genus (in the family Rhodospirillaceae), the Amoebophilaceae and Flavobacteriaceae families, and the Alphaproteobacteria class. In the case of *Inquilinus* and Flavobacteriaceae, the clone libraries captured multiple sequences corresponding to those OTUs with at least 97% identity.

**Table 3.** Alignment statistics for the core next-generation bacterial OTUs that were captured by clone library sequences. Clone library OTUs are included in this table if the BLASTN result showed query cover ≥ 90% and identity ≥ 97%. Length of all next-generation sequences was 253 bp. Source of next-generation sequence data is Sharp et al. [5].

Greengenes classification of next-generation OTU (to lowest level)	Next-gen OTU identifier	Corresponding clone library OTU identifier	Clone library sequence accession number	Length of clone library sequence	Identity between sequences in aligned portion	Gaps
*Inquilinus*	301588	NCUR_OTU46	MK176023	823 bp	253 bp/253 bp (100%)	0/253 (0%)
*Inquilinus*	301588	NCUR_OTU203	MK175889	866 bp	249 bp/253 bp (98%)	0/253 (0%)
Amoebophilaceae	590468	NCUR_OTU241	MK175907	841 bp	253 bp/253 bp (100%)	0/253 (0%)
Flavobacteriaceae	807522	689465	MK175665	828 bp	247 bp/253 bp (98%)	0/253 (0%)
Flavobacteriaceae	807522	2676455	MK175756	858 bp	247 bp/254 bp (97%)	1/254 (0%)
Flavobacteriaceae	807522	NCUR_OTU817	MK176202	823 bp	245 bp/253 bp (97%)	0/253 (0%)
Alphaproteobacteria	4313721	NCUR_OTU91	MK176254	831 bp	253 bp/253 bp (100%)	0/253 (0%)

The next-generation sequencing by Sharp et al. also identified four archaeal OTUs that appeared in all eight of the samples analyzed here. Only two of the eight samples were used for archaeal sequencing in this study, but all four of the OTUs were detected among those two clone libraries (FW1B8 and FW1W8). Comparing each next-generation archaeal OTU to its clone library counterpart demonstrated close alignment (Table 4).

**Table 4.** Alignment statistics for the four archaeal OTUs appearing both in clone libraries and in next-generation sequences. All next-generation OTU sequences were 253 bp in length. All OTUs were classified by Greengenes to the class Thaumarcheota, genus *Nitrosopumilus*. Source of next-generation sequence data is Sharp et al. [5].

OTU identifier (from Greengenes)	Clone library sequence accession number	Length of clone library sequence	Portion of next-generation sequence covered in alignment	Gaps in next-generation sequence upon alignment	Identity between sequences in aligned portion
152483	MH915526	782 bp	251 bp/253 bp	0	99%
154353	MH915527	874 bp	252 bp/253 bp	0	99%
461312	MH915530	872 bp	253 bp/253 bp	0	98%
4369009	MH915528	854 bp	253 bp/253 bp	1	98%

## 4. Discussion

Every bacterial class (n = 10) detected whose members constituted at least 2.5% of the bacterial community of at least one sample in the Illumina data set was also detected here in the clone library data set as at least 2.5% of at least one sample's bacterial community. Three additional classes (Verrucomicrobiae, Nitrospira, Betaproteobacteria) comprised at least 2.5% of the bacterial community of at least one clone library sample, but not of an Illumina sample (Figure 1). By both sequencing methods, Proteobacteria were the dominant bacterial class in every *A. poculata* sample. Moreover, the same five bacterial classes comprised the highest mean portion of *A. poculata* communities based on clone libraries (5.0% to 35.8%) as well as Illumina sequencing (4.6% to 29.5%). Thus, the bacterial communities exhibited overall stability regardless of the sequencing platform used to examine the diversity within those communities.

There are several factors that may have contributed to the high level of similarity observed between the results of the Illumina study and the present results. This study used the same DNA extractions as in Sharp et al. [5], eliminating extraction method as a potential source of variability in community composition. The choice of 16S region to amplify can play a role in determining the relative abundance of bacterial community members [33], but in the present study, the region sequenced entirely encompassed the V4 region sequenced in the Illumina study. Thus, although different primers and sequencing platforms can affect the observed relative abundance of microbiome members [34], the use of the same samples, extractions, and sequenced regions, combined with the stability of the *A. poculata* microbiome, likely contributed to the resemblance detected in bacterial community composition between the studies.

The reason that the samples yielded greater diversity through next-generation sequencing than through clone library sequencing (Table 2) is likely due to the greater number of sequences generated by the Illumina method. Even after rarefaction, 3516 next-generation sequences remained for each sample, while the number of clone library sequences analyzed for each sample ranged from 46 to 326 (Table 1). Higher alpha diversity indices associated with next-generation sequencing were also described in a study of the cervical microbiome comparing microbial diversity obtained by three different sequencing methods (Sanger, Illumina, and 454 pyrosequencing) [35]. The Shannon index yielded by Illumina sequencing was consistently higher than the same measure when determined from Sanger sequencing. The differences between the Shannon index for the sequencing methodologies illustrate one of the primary advantages of using next-generation sequencing methods: generation of a greater number of sequences, by an order of magnitude, than can be cost-effectively obtained through clone libraries.

Beta diversity is usually stable between data sets obtained by Sanger and Illumina sequencing, and thus the sequencing method should not affect community comparisons [36],[37]. Nelson et al. [38] agree that beta diversity is less affected by sequencing platform than alpha diversity, although Gihring et al. [39] caution against comparisons between studies that used different numbers for rarefying sequence sets, given that sample size influences diversity estimates. In accordance with that view, Sharp et al.'s [5] Illumina sequences from *A. poculata* would ideally be rarefied to the same number as the clone library sequences in this study in order to compare the beta diversity calculated in each study. However, that would mean eliminating more than 98% of the information provided by the next-generation sequences. Therefore, the differences in rarefaction depth (3516 for next-generation sequences, but 46 for Sanger sequences) likely explain the differences we observed in beta diversity, in which season made a difference in the Illumina data set, but not in the Sanger data set.

The bacterial classes identified in the community composition analysis as meeting a 2.5% threshold appear as large colored clusters on the phylogenetic tree (Figure 2). Those classes are well represented by both sequencing methods. These results demonstrate that members of the microbiome identified as dominant by next-generation sequencing are also likely to be identified through clone library sequencing, although there are many more representatives of next-generation OTUs within those classes. Some of these additional OTUs may be genuine detections due to the increase in sequencing depth. However, Illumina MiSeq's most common source of error is substitution type miscalls [40]. Studies using mock communities have demonstrated that common methods of alignment of MiSeq sequences for OTU clustering can then compound the sequencing errors by predicting many more OTUs than actually exist [41]–[43].

Clone library sequencing of *A. poculata*'s bacterial community captured all four core bacterial OTUs previously identified by Sharp et al. [5] (Table 3). Moreover, one of the next-generation OTUs (OTU 807522, from the Flavobacteriaceae family) constituted less than 2.4% of the microbiome of one of the samples examined here, and less than 1.1% of the other seven samples. The other core OTUs represented 0.1% to 8.5% of the samples (OTU 4313721, Alphaproteobacteria), 0.05% to 5.4% of the samples (OTU 590468, Amoebophilaceae), and 0.02% to 6.8% of the samples (OTU 301588, *Inquilinus*). Taken together, this indicates that clone library sequencing can still capture relatively rare bacterial taxa within a microbial community.

Because this study could not capture archaeal sequences at the same time as bacterial sequences, but instead had to amplify and clone those sequences separately, we cannot draw a quantitative conclusion regarding percentages of the prokaryotic community. However, it is worth noting that a substantial amount of sublevel archaeal diversity was discovered. Archaeal sequences were distributed into 18 OTUs, 16 of which were classified by Greengenes as the *Nitrosopumilus* genus (part of the Cenarchaeaceae family). The other two OTUs were classified only as far as the family level, into the Cenarchaeaceae family. The archaeal OTUs revealed by clone library sequencing included all four archaeal OTUs identified by Sharp et al. [5] as occurring in all eight of the samples analyzed here (Table 4). As in the results of the bacterial clone libraries, these results establish that prevalent members of the archaeal community detected by next-generation sequencing are also likely to be identified by clone libraries.

## 5. Conclusions

This study examined the microbiomes of eight colonies of the temperate coral *Astrangia poculata*, in order to determine whether fewer, longer sequences obtained by Sanger sequencing could capture key diversity (e.g., dominant and core members of the microbiome) as identified by shorter reads previously produced by the Illumina platform. Microbiome diversity was stable and remarkably similar across the two sequencing platforms. The primary taxa identified by Sanger sequencing were the same as those revealed by Illumina sequencing. Moreover, the sequences obtained in this study included the five OTUs (four bacterial and one archaeal) identified as core to *A. poculata* by Sharp et al. [5]. Minor differences in the relative abundance of community members could be attributable to the different sequencing platforms, and could also arise from biases produced by the use of different primer sets and rarefaction depths. However, phylogenetic analysis demonstrated that the Sanger sequences substantially overlapped with the Illumina sequences from the *A. poculata* microbiomes. Thus, this study demonstrates that Sanger sequencing was capable of reproducing the biologically-relevant diversity detected by deeper next-generation sequencing, while also producing longer sequences useful to the research community for probe and primer design.
